# Unified 0.25-degree gridded infrastructure-critical extreme weather for the United States from 1979 to 2100

**DOI:** 10.1038/s41597-025-05918-5

**Published:** 2025-09-12

**Authors:** Tao Sun, Chad Zanocco, June Flora, Aditi Sheshadri, Ram Rajagopal

**Affiliations:** 1 Department of Civil & Environmental Engineering, Stanford University, Stanford, CA USA; 2 Department of Earth System Science, Stanford University, Stanford, CA USA; 3 Department of Electrical Engineering, Stanford University, Stanford, CA USA

**Keywords:** Climate and Earth system modelling, Natural hazards

## Abstract

Extreme weather events can severely disrupt critical infrastructure, triggering cascading effects on power, transportation, and essential services. However, standard weather and climate datasets often lack specialized variables necessary for hazard assessments. We present a unified dataset of infrastructure-critical weather and climate variables across the United States at 0.25° resolution, covering daily or sub-daily intervals from 1979 to 2100. The dataset includes temperature, dew point, wind gusts, precipitation partitioned by rain, snow, and freezing rain or ice pellets, lightning, and wildfire metrics. Historical conditions (1979-2023) are synthesized from observations and reanalysis products, while future projections are derived from 14 CMIP6 global climate models (historical, SSP245, and SSP585 experiments). Physically based and data-driven methods are used to estimate variables not directly provided by existing models. By integrating these variables into a single unified dataset, we enable consistent, high-resolution assessments of weather-related infrastructure risks across past and future periods, supporting wide-ranging applications in energy, transportation, water resources, emergency management, and beyond.

## Background & Summary

Infrastructure systems are frequently exposed to hazardous weather conditions, which can create cascading disruptions threatening both essential community functions and the health, safety and well-being of individuals^1–3^. For instance, strong wind gusts can damage electrical grids and disrupt power to medical facilities, water treatment plants, and heating systems^4^. Freezing rain rapidly ices roads and railways, and can trigger transportation accidents and supply chain failures^5^. Wildfires can devastate critical infrastructure and contaminate air and water resources over wide geographic extents^6^, while heatwaves can strain power grids beyond capacity and cause materials to warp and fail^7^. Intense lightning strikes can spark wildfires or damage sensitive power and communication equipment^8,9^. Collectively, these hazards are already imposing substantial socioeconomic costs, public health emergencies, and safety threats—impacts that are projected to intensify under a changing climate.

Despite the growing urgency of weather- and climate-driven infrastructure vulnerabilities, datasets capturing historical records of these hazards remain fragmented. Reanalysis products, such as ECMWF Reanalysis v5 (ERA5)^10^ and North American Regional Reanalysis (NARR)^11^, supply certain variables but lack others, such as lightning flash rates or wildfire metrics. Specialized observation networks, including National Lightning Detection Network (NLDN)^12^ for lightning and various wildfire archives^13–17^, provide high-quality coverage of individual hazards but often use disparate formats and heterogeneous spatial/temporal resolutions, complicating multi-hazard assessments. These gaps hamper efforts to build a unified view of historical infrastructure-relevant hazards and make it difficult to examine interactions or cascading failures across multiple threats.

Deficiencies also persist when projecting these hazards under future climate conditions. Widely used global modeling efforts for future climate such as Coupled Model Intercomparison Project Phase 6 (CMIP6)^18,19^ provide meteorological fields such as temperature and precipitation, but do not directly produce specialized infrastructure-critical variables. Meanwhile, there is literature that derives wind gusts^20–22^, freezing rain^23–28^, snowfall^29–33^, lightning^34,35^, wildfires^36–38^ from these standard fields in climate modeling, yet these studies and datasets often face several limitations. Some rely on coarse spatial grids (e.g., 1°–3°) that may not sufficiently capture local-scale variability crucial for infrastructure impacts, whereas others may have low temporal resolution (e.g., monthly timesteps), that misses the daily or sub-daily spikes most relevant for failure thresholds. Geographic coverage may also be narrow, such as a single state or region, limiting the scope of broader analyses. In some cases, there is no open data access, reducing applicability for broader scientific and policy efforts. Certain approaches re-run climate models at specialized resolution to obtain non-standard outputs, but typically only incorporate a single or small set of global climate models (GCMs), thus failing to capture the ensemble-based stochasticity that is vital for robust climate projections and scenario development. Moreover, many of these datasets differ in spatiotemporal resolution and extent, hampering efforts to assess multi-hazard interactions—an essential consideration, given that infrastructure systems are often exposed to multiple concurrent threats. While some studies^39–41^ offer multiple climate extreme indices in a unified framework, these indices are typically derived directly from standard GCM outputs like temperature or precipitation and do not include specialized, infrastructure-critical variables.

To address these limitations, we present a unified, comprehensive gridded dataset of infrastructure-critical variables at 0.25° resolution, spanning both historical records (1979-2023) and future scenarios (to 2100). This dataset (see Table 1) includes daily or sub-daily wind gusts, temperature and dew point, precipitation by type (freezing rain or ice pellets, snow, and rain), lightning flash rates, and wildfire metrics, all harmonized in a consistent format. The dataset covers all 50 U.S. states and the District of Columbia, with the exception of historical lightning data for Hawaii due to NLDN coverage limitations. Historical fields blend observational and reanalysis sources, while future projections are derived from 14 CMIP6 models^42–55^ using physically based and data-driven methods to infer variables not directly produced by the models. The pipeline for generating the resulting dataset can be updated with newly available climate simulations or improved reference data, facilitating robust, ensemble-based assessments of infrastructure risks across multiple weather hazards. Users should note that our dataset employs different observational baselines (for example, ERA5, NARR) than those used in the National Climate Assessments (NCAs)^56^, which rely on Localized Constructed Analogs (LOCA) downscaling^57,58^ based on Livneh historical observations^59^. This difference may lead to discrepancies in overlapping variables like temperature and precipitation, and users integrating our dataset with existing NCA-like assessments should account for these potential inconsistencies (literature comparing observational datasets^60^ could provide additional context for this).

**Table 1:** List of infrastructure-critical variables in this dataset.

Variable	Description	Frequency	Unit
fg10	Maximum near-surface wind gust	6hr^−1^	m ⋅ s^−1^
tas	Near-surface air temperature	day^−1^	degrees K
tds	Near-surface dew point temperature	day^−1^	degrees K
ta	Air temperature on pressure levels	day^−1^/3hr^−1^	degrees K
pfrz	Accumulated total precipitation from freezing rain or ice pellets	day^−1^	kg ⋅ m^−2^
psnow	Accumulated total precipitation from snow	day^−1^	kg ⋅ m^−2^
prain	Accumulated total precipitation from rain	day^−1^	kg ⋅ m^−2^
flashrate	Frequency of lightning flashes per unit area	day^−1^	km^−2^ ⋅ day^−1^
fburn	Binary indicator of fire actively burning	day^−1^	dimensionless
fpop	Fraction of population affected by fires	day^−1^	fraction
fline	Fraction of power transmission lines affected by fires	day^−1^	fraction

Note: The ta field is excluded from the online repository because of its storage size (several TBs). It serves only as an intermediate variable in our processing workflow and can be regenerated with the accompanying code.

## Methods

This section details how we derived each variable in our unified dataset for both the historical period and future climate scenarios at 0.25° spatial resolution with daily or sub-daily timesteps. Historical conditions come from observational or reanalysis products (ERA5, NARR, NLDN, and multiple wildfire archives), each originally at different resolutions and thus regridded to a common 0.25° grid. Future conditions rely on 14 CMIP6 GCMs (under the historical simulation, SSP245, and SSP585) and their downscaled products, NASA Earth Exchange Global Daily Downscaled Projections for CMIP6 (NEX-GDDP-CMIP6)^61^. Many target variables (e.g., wind gusts, freezing rain, lightning, wildfire) are not directly provided by GCMs, so we infer them via data-driven or physically based methods from the model fields that are available. Because not all GCMs offer the key inputs (e.g., daily maximum wind speed, pressure-level temperatures, or 3-hourly temperature) necessary for these derivations, we select the 14 models that do supply them across the required experiments and temporal coverage; Table 2 lists the specific GCMs used. It should be noted that approximately one-fifth of CMIP6 models exhibit equilibrium climate sensitivity (ECS) exceeding 5°C^62^, and within our selection, HadGEM3-GC31-LL and UKESM1-0-LL fall into this “hot model” category^63^, which may influence projections of temperature-dependent variables. While these models project higher warming scenarios, they may provide valuable bounds for assessing and preparing for extreme events. Although ERA5 and NEX-GDDP-CMIP6 both nominally adopt a 0.25° grid, their grid cell centers are offset by 0.125°, resulting in a grid alignment difference. We adopt the NEX-GDDP-CMIP6 grid as our standard, regridding other datasets when necessary. To ensure consistent observational references, we generally apply bias correction to original CMIP6 model outputs and NEX-GDDP-CMIP6 variables against our observational baselines (ERA5, NARR, etc.), despite NEX-GDDP-CMIP6 having undergone an initial bias correction on another baseline^64^, because differences in chosen observational data can influence the final corrected fields^65^. The subsections below describe in detail how we obtain wind gusts, temperature and dew point, vertical temperature profiles, precipitation by type, lightning flash rates, and wildfire metrics.

**Table 2:** GCMs used in this dataset.

No.	Name	Institution/center	Original approx. resolution (Longitude × Latitude)	Variant
1	BCC-CSM2-MR	BCC	~1.12° × 1.12°	r1i1p1f1
2	CMCC-CM2-SR5	CMCC	~1.12° × 0.94°	r1i1p1f1
3	CMCC-ESM2	CMCC	~1.25° × 0.94°	r1i1p1f1
4	CNRM-CM6-1	CNRM	~1. 4° × 1. 4°	r1i1p1f2
5	CNRM-ESM2-1	CNRM	~1. 4° × 1. 4°	r1i1p1f2
6	EC-Earth3	EC-Earth Consortium	~1.125° × 1.125°	r1i1p1f1
7	INM-CM4-8	INM	~2. 0° × 1. 5°	r1i1p1f1
8	INM-CM5-0	INM	~2. 0° × 1. 5°	r1i1p1f1
9	MIROC6	MIROC	~1. 4° × 1. 4°	r1i1p1f1
10	MPI-ESM1-2-HR	MPI-M	~0. 9° × 0. 9°	r1i1p1f1
11	MRI-ESM2-0	MRI	~1.125° × 1.125°	r1i1p1f1
12	HadGEM3-GC31-LL	Met Office Hadley Centre	~1.875° × 1.25°	r1i1p1f3
13	KACE-1-0-G	NIMR/KMA	~1.875° × 1.25°	r1i1p1f1
14	UKESM1-0-LL	Met Office Hadley Centre/UKESM	~1.875° × 1.25°	r1i1p1f2

### Wind gust (0.25°, 6-hourly)

We produce 6-hourly maximum near-surface wind gusts (fg10) at 0.25° resolution for both the historical period (1979-2023) and future projections through 2100. Short-lived wind gusts, rather than sustained winds, can impose the largest strain on infrastructure; hence our focus on gust peaks at sub-daily time steps.

#### Historical period (1979-2023)

For the historical dataset, we draw on ERA5 reanalysis at 0.25° resolution, which provides hourly fg10, as well as the hourly eastward (u10) and northward (v10) near-surface wind speed components. From hourly u10 and v10, we compute hourly near-surface wind speed (ws10) as the Euclidean magnitude $\sqrt{u1{0}^{2}+v1{0}^{2}}$. To develop 6-hourly fg10, we identify the maximum fg10 value in each 6-hour window, producing a 6-hourly series of peak gusts. In addition to these 6-hourly gusts, we calculate daily ws10 by averaging hourly ws10 across each 24-hour day, and daily maximum wind speed (ws10max) by taking the highest hourly ws10 within that day. These ERA5 wind variables were regridded using bilinear interpolation to align with the NEX-GDDP-CMIP6 grid (also 0.25° resolution). These daily fields serve as the foundation for subsequent bias corrections and for establishing the statistical relationships needed to project future gusts.

#### Future projections (through 2100)

For future climate scenarios, we employ outputs from 14 CMIP6 GCMs (coarse resolution  ~1°–3°) that provide daily ws10 (sfcWind in CMIP6) and ws10max (sfcWindmax in CMIP6). We also incorporate the NEX-GDDP-CMIP6 dataset at 0.25°, which includes daily ws10 but not ws10max. We apply a monthly quantile-mapping bias correction to these variables over the 1979-2014 reference period. Specifically, we first regrid (conservative) ERA5 daily ws10 and ws10max to each GCM’s native resolution, then bias-correct the GCMs’ daily ws10 and ws10max against the corresponding ERA5 fields. The same procedure is applied to NEX-GDDP-CMIP6’s daily ws10, referencing ERA5 at 0.25° resolution.

We generate 6-hourly fg10 for these future scenarios using the k-nearest neighbors approach^66^ (k=1) trained on historical ERA5 (1979-2014) for each GCM. Conceptually, the k=1 nearest neighbor approach places each future day’s characteristics into a multi-dimensional feature space and finds the single historical day in ERA5 whose features are most similar (i.e., nearest) in that space. We build each model’s training set from “day-(0.25°) cell” combinations in ERA5, regridding (conservative) ERA5 data to each GCM’s native resolution as needed. For each future day and 0.25° grid cell, we identify a “historically similar” day-location in ERA5 by matching four features:

- daily ws10 at the nearest coarse GCM grid cell,
- the ratio of daily ws10max to ws10 at that coarse grid cell,
- the ratio of daily ws10 at the 0.25° cell to ws10 at the coarse grid cell,
- the latitude-longitude of the 0.25° cell (ensuring the best match is geographically close).

From the best-matching historical day-location, the nearest neighbor model retrieves the ratios of 0.25° cell’s 6-hourly fg10 to the nearest coarse grid cell’s daily ws10. These ratios are then multiplied by the examined future day’s daily ws10 at the nearest coarse grid cell, producing an initial 6-hourly fg10 estimate for the examined 0.25° cell. Finally, we apply a monthly quantile-mapping (referencing ERA5 at 0.25° resolution for 1979-2014) to the resulting 6-hourly fg10, ensuring their distribution remains consistent with historical variability. In this approach, all days within each month are adjusted using the same quantile mapping function derived specifically for that month.

### Temperature & dew point (0.25°, daily)

We produce daily near-surface air temperature (tas) and dew point temperature (tds) at 0.25° resolution for both the historical period (1979-2023) and future projections through 2100. These fields support analyses of heat or cold extremes, moisture conditions, and derived metrics such as cooling and heating degree days.

#### Historical period (1979-2023)

For the historical dataset, we draw on ERA5 reanalysis at 0.25° resolution, which provides daily near-surface temperature (t2m) and near-surface dew point temperature (d2m). For consistency, we refer to these variables as tas and tds, respectively. These ERA5 fields are regridded using bilinear interpolation to align with the NEX-GDDP-CMIP6 grid (also 0.25° resolution) and also form the baseline for characterizing historical temperature and humidity conditions and for bias-correcting future model outputs.

#### Future projections (through 2100)

For future climate scenarios, we obtain daily tas and relative humidity (hurs) from the same 14 GCMs, using 0.25° downscaled outputs in NEX-GDDP-CMIP6. To derive tds, we first convert tas (in K) to Celsius (*T*_C_ = tas − 273.15) and express hurs as a fraction (hurs/100). We then compute the saturation vapor pressure (*e*_*s*_) via a Magnus-type formula,

1 $${e}_{s}=6.112\,\exp (\frac{17.67\,{T}_{C}}{{T}_{C}+243.5}),$$

where 6.112 hPa, 17.67, and 243.5 °C are empirical constants. Multiplying *e*_*s*_ by hurs/100 yields the actual vapor pressure (*e*). The dew point temperature in Celsius, *T*_dew,C_, is then obtained by inverting the same relation,

2 $${T}_{dew,C}=\frac{243.5\,ln(\frac{e}{6.112})}{17.67\,-\,ln(\frac{e}{6.112})},$$

and subsequently converted back to tds (in K). Both daily tas and this derived tds undergo monthly quantile mapping (reference period: 1979-2014) against ERA5’s corresponding fields, ensuring that their distributions align with historical variability. Due to the nature of temperature fields, this monthly quantile mapping may introduce minor discontinuities at month boundaries.

### Temperature profiles at pressure levels (0.25°, daily and 3-hourly)

We produce daily and 3-hourly temperature fields at 0.25° resolution, spanning pressure levels from 1000 hPa down to 100 hPa, for both the historical period (1979-2014) and future projections through 2100. These vertical profiles are instrumental for precipitation-type classification, lightning-flash modeling, and other meteorological analyses sensitive to aloft temperature structures. Two complementary datasets are provided: (a) a daily product at 100 hPa increments from 1000 hPa to 100 hPa and (b) a 3-hourly product at 50 hPa increments over the same pressure range.

#### Historical period (1979-2014)

For the historical dataset, we draw on ERA5 reanalysis at 0.25° resolution, which provides hourly temperature profiles (1000-100 hPa in 50 hPa increments) and hourly tas (t2m in ERA5). We form daily profiles by averaging these hourly data over 24-hour periods, and generate 3-hourly profiles by averaging over 3-hour windows. We also compute 3-hourly tas from hourly tas using the same time-averaging procedures. Again, these ERA5 fields are regridded using bilinear interpolation to align with the NEX-GDDP-CMIP6 grid (also 0.25° resolution). Because these historical temperature profile data primarily serve as intermediate inputs (e.g., for training the data-driven model), we provide historical coverage only until 2014.

#### Future projections (through 2100)

For future climate scenarios, we use outputs from 14 CMIP6 GCMs, which typically provide daily and 3-hourly tas as well as daily temperature profiles at a limited set of pressure levels (1000, 850, 700, 500, 250, 100 hPa) at coarse resolution (~1°–3°). We also incorporate the 0.25° NEX-GDDP-CMIP6 dataset, which offers daily tas but does not include aloft temperature fields. To ensure consistency with ERA5, we first regrid (conservative) ERA5 daily tas to each GCM’s native resolution and apply monthly quantile mapping (reference period: 1979-2014) to bias-correct each GCM’s daily tas. We similarly bias-correct the NEX-GDDP-CMIP6 daily tas, referencing ERA5’s daily tas at 0.25°. We do not bias-correct the GCM’s temperature at pressure levels or its 3-hourly tas, since modifying those fields can disrupt the vertical and temporal relationships essential for precipitation-type classification and lightning-flash modeling applications. Users should note that this may introduce inconsistencies between temperature profiles and near-surface temperatures; while suitable for applications dependent on either temperature profiles alone or near-surface temperatures alone, caution is advised for applications requiring consistency between both.

To reconstruct daily temperature profiles at 0.25°, we train a separate k-nearest neighbors (k = 1) model for each GCM on historical ERA5 data (1979-2014). We build the model’s training set from “day–(0.25°) cell” combinations in ERA5, regridding (conservative) ERA5 data to each GCM’s native resolution where necessary. For each future day and 0.25° cell, we identify a “historically similar” day-location in ERA5 by matching four features:

- daily tas at the nearest coarse GCM grid cell, *T*_ns,GCM_,
- the ratio of each pressure-level temperature (*T*_*p*,GCM_) at *p* = 850, 700, 500, 250, 100 hPa to the temperature at 1000 hPa (*T*_1000,GCM_) at that same coarse GCM grid cell,
- the ratio of daily tas at the 0.25° cell (*T*_ns,0.25_) to *T*_ns,GCM_,
- the latitude-longitude of the 0.25° cell.

From the best-matching historical day-location in ERA5, the model retrieves the ratio (*T*_*p*,0.25_/*T*_ns,GCM_) spanning the six discrete pressure levels (1000, 850, 700, 500, 250, 100 hPa), where *T*_*p*,0.25_ is the aloft temperature at pressure *p* at 0.25°. Multiplying these ratios by the future day’s *T*_ns,GCM_ yields daily temperature profiles at the same six pressure levels for the future day-location of interest. We then linearly interpolate between these six levels to obtain a complete profile in 100 hPa increments from 1000 hPa down to 100 hPa.

We generate 3-hourly temperature profiles at 0.25° by extending the nearest neighbor approach to include sub-daily features. Specifically, for each future day and 0.25° cell, we match five inputs to find a “historically similar” day-location in ERA5:

- daily tas at the nearest coarse GCM grid cell, *T*_ns,GCM_,
- the ratio of each 3-hourly tas $({T}_{ns,GCM}^{j})$ (for *j* = 1, …, 8) to that day’s average 3-hourly tas at that same coarse GCM grid cell,
- the ratio of daily tas at the 0.25° cell (*T*_ns,0.25_) to *T*_ns,GCM_,
- the daily aloft-temperature ratios (*T*_*p*,0.25_/*T*_ns,GCM_) at the six pressure levels (1000, 850, 700, 500, 250, 100 hPa) obtained from the daily nearest neighbor model,
- the latitude-longitude of the 0.25° cell.

From this best match, the model retrieves $({T}_{p,0.25}^{j}/{T}_{ns,GCM})$ at 50 hPa increments between 1000 and 100 hPa, where ${T}_{p,0.25}^{j}$ is the aloft temperature at pressure level p and 3-hour slot *j* at 0.25^∘^. Multiplying these ratios by the future day’s *T*_ns,GCM_ produces 3-hourly temperature profiles at 0.25^∘^, covering 1000-100 hPa in 50 hPa increments.

### Precipitation by type (0.25°, daily)

We produce daily precipitation amounts for freezing rain or ice pellets (pfrz), snowfall (psnow), and rain (prain) at 0.25° resolution for both the historical period (1979-2023) and future projections through 2100. Our approach begins by classifying precipitation every three hours to capture sub-daily variations in precipitation type, after which we aggregate these sub-daily amounts into daily totals for each category. This procedure ensures a clear partitioning of freezing rain or ice pellets, snowfall, and rain at a resolution relevant to infrastructure-impact analyses.

#### Historical period (1979-2023)

We use the NARR, which provides 3-hourly precipitation amounts (apcp, in kg ⋅ m^−2^) on a 32 km (~0.3°) Northern Lambert Conformal Conic grid. NARR also includes 3-hourly categorical fields identifying freezing rain (cfrzr), ice pellets (cicep), snow (csnow), and rain (crain). By merging these flags with the 3-hourly precipitation amounts, we derive precipitation in each category, then sum to daily totals to obtain pfrz, psnow, and prain. We regrid (nearest neighbor) these data to a uniform 0.25° latitude-longitude domain. Here, we group freezing rain and ice pellets as a single category, reflecting their closely related atmospheric formation processes and similar hazardous impacts on infrastructure. Users should note that because we employ NARR for precipitation while using ERA5 for other variables such as temperature and humidity, potential inconsistencies between these reanalysis datasets^67,68^ may occasionally result in physically implausible combinations, such as precipitation occurring with incompatibly low humidity or precipitation types that do not align with concurrent temperature conditions.

#### Future projections (through 2100)

For future scenarios, we draw on precipitation rate (pr, in kg ⋅ m^−2^ ⋅ s^−1^) outputs from 14 CMIP6 GCMs: twelve models provide 3-hourly pr on coarse grids (~1°–3°), while the remaining two supply only daily pr. We also incorporate daily pr at 0.25° from NEX-GDDP-CMIP6. For each day, GCM, and 0.25° grid cell, we form the ratio of the 0.25° daily pr to the daily pr from the nearest coarse GCM cell, thereby aligning coarse GCM precipitation to the 0.25° grid while preserving the daily total dictated by the downscaled product. Where 3-hourly GCM data are available, each 3-hour pr is multiplied by this ratio to yield 3-hourly pr at 0.25°. If 3-hourly GCM data are not available, we interpret the daily pr uniformly over eight 3-hour intervals, assigning one-eighth of the 0.25° daily total precipitation amount to each 3-hour timestep. This procedure ensures a consistent 3-hourly precipitation series at 0.25°. For consistency with the historical period data, we then convert these 3-hourly pr (kg ⋅ m^−2^ ⋅ s^−1^) at 0.25° to 3-hourly apcp (kg ⋅ m^−2^) by multiplying each rate by 10,800 s (3 hours).

##### Precipitation-type classification (3-hourly)

We classify precipitation type at 0.25° resolution by combining the 3-hourly apcp with 3-hourly temperature profiles (1000-100 hPa in 50 hPa increments), following the Bourgouin method^69,70^. This approach identifies “positive” (warm) areas (PA), where above-freezing temperatures can melt solid precipitation, and “negative” (cold) areas (NA), where sub-freezing temperatures can refreeze liquid precipitation. To compute these areas, we use the thermodynamic relation:

3 $$Area\,=\,{c}_{p}\,\overline{{T}_{l}}\,ln(\frac{{\theta }_{top}}{{\theta }_{bottom}}),$$

where *c*_*p*_ ≈ 1004 J kg^−1^ K^−1^ is the specific heat at constant pressure, $\overline{{T}_{l}}$ is the mean temperature of the layer, and *θ*_top_, *θ*_bottom_ are the potential temperatures at the top and bottom of the layer. These potential temperatures are defined by

4 $${\theta }_{top}\,=\,{T}_{top}{(\frac{{P}_{0}}{{P}_{top}})}^{\frac{{R}_{d}}{{c}_{p}}},\quad {\theta }_{bottom}\,=\,{T}_{bottom}{(\frac{{P}_{0}}{{P}_{bottom}})}^{\frac{{R}_{d}}{{c}_{p}}},$$

where *T*_top_ = *T*_bottom_ = 273.15 K marks the 0 °C transition, *P*_0_ = 1000 hPa is the reference pressure, *R*_*d*_ ≈ 287 J kg^−1^ K^−1^ is the gas constant for dry air, and *P*_top_, *P*_bottom_ are the pressures bounding the layer of interest. Under these conditions, the expression simplifies to

5 $$Area\,=\,{R}_{d}\,\overline{{T}_{l}}\,ln(\frac{{P}_{bottom}}{{P}_{top}}).$$

Evaluating PA and NA in this manner reveals potential melting and refreezing layers, allowing us to classify each 3-hour timestep as snow, freezing rain or ice pellets, or rain according to Bourgouin’s criteria. Specifically:

- Warm layer above a cold surface layer: freezing rain/ice pellets if PA ≥ 2 J kg^−1^, snow if PA < 2 J kg^−1^.
- Surface-based warm layer only: snow if PA ≤ 13.2 J kg^−1^, rain if PA > 13.2 J kg^−1^.
- One cold area and two warm layers (letting the aloft warm layer have PA_1_ and the surface-based warm layer have PA_2_):
  - Snow if PA_1_ < 2 J kg^−1^ and PA_2_ ≤ 13.2 J kg^−1^.
  - Rain if PA_1_ < 2 J kg^−1^ and NA < (46 J kg^−1^ + 0.66 × PA_1_).
  - Freezing rain/ice pellets if PA_1_ ≥ 2 J kg^−1^, NA ≥ (46 J kg^−1^ + 0.66 × PA_1_), and PA_2_ ≤ 13.2 J kg^−1^.
- No warm layer: all precipitation remains snow.

##### Bias correction and daily totals by precipitation type

Having classified precipitation types at each 3-hour step, we aggregate the 3-hourly amounts into daily totals for (a) pfrz, (b) psnow, and (c) prain at each 0.25° cell. We then apply a type-specific bias correction via quantile mapping for the daily precipitation amounts in each category, referencing historical NARR distributions (1979-2014).

Through this sub-daily classification, daily aggregation, and subsequent bias correction, our dataset provides 0.25° precipitation by type under both current and future climate conditions—facilitating a wide range of impact analyses related to winter-weather hazards, hydrologic extremes, and infrastructure resilience.

### Flash lightning (0.25°, daily)

We produce a daily lightning flash rate (flashrate) dataset at 0.25° resolution for both the historical period (1987-2023) and future scenarios extending to 2100. Lightning poses substantial risks for infrastructure and offers important insights into convective dynamics under a changing climate. The historical dataset is derived from observed cloud-to-ground flashes, whereas the future projections rely on a Convective Available Potential Energy (CAPE)-based modeling approach that links lightning occurrence to thermodynamic and precipitation variables in CMIP6 outputs.

#### Historical period (1987-2023)

For the historical record, we obtain daily lightning flash counts from the NLDN, accessed via the Severe Weather Data Inventory (SWDI). These counts, available on a 0.10° grid for the continental United States, are summed daily and then normalized per square kilometer (counts ⋅ km^−2^ ⋅ day^−1^). We subsequently regrid (conservative) these data to a uniform 0.25° grid, preserving total flash counts. Simultaneously, we acquire daily CAPE at  ~32 km (0.3°) resolution from NARR, which is also regridded (nearest neighbor) to 0.25° to align with the lightning data. The resulting historical record of daily flashrate and CAPE (1987-2023) serves both as a standalone dataset and as baseline data for future projections.

#### Future projections (through 2100)

Direct daily lightning outputs are rarely available from CMIP6 GCMs, so we adopt an established relationship^34,35,71^ in which flashrate is correlated with the product of CAPE and precipitation (CAPE × apcp). A commonly used parameterization takes the power-law form

6 $$flashrate\,=\,a\,{(CAPE\times apcp)}^{b},$$

where flashrate is in counts ⋅ km^−2^ ⋅ day^−1^, CAPE in J ⋅ kg^−1^, and apcp in kg ⋅ m^−2^. This can also be expressed in log-linear form:

7 $$ln(flashrate)\,=\,b\,ln(CAPE\times apcp)\,+\,ln(a),$$

which allows parameters *a* and *b* to be estimated via linear regression on the log-transformed variables. Typically, such an estimation is performed at monthly or coarser temporal scales, and over larger spatial domains, to ensure more stable parameter fits. However, at daily and 0.25° resolution, lightning data typically exhibit numerous zero-flash days and sporadic extremes, complicating simple linear regression approaches. We therefore employ a quantile-regression framework that (i) extends the log-linear model to

8 $$ln(flashrate)\,=\,{b}_{1}\,ln(CAPE)\,+\,{b}_{2}\,ln(apcp)\,+\,ln(a),$$

and (ii) accommodates extremes and zero-inflated conditions by fitting conditional quantiles instead of just the mean under linear regression. This yields a more robust depiction of lightning’s stochastic nature.

We train this model using daily historical data (1987-2023) at 0.25° resolution across the United States, restricting the sample to day-grid cells with CAPE > 0 or apcp > 0 because lightning rarely occurs otherwise. After establishing the quantile-regression relationships, we then apply them to future CAPE and apcp under the 14 CMIP6 GCMs, noting that CAPE is not directly provided by these GCMs and must be computed at 0.25° resolution (as described below). For daily apcp under future scenarios, we use the 0.25° downscaled (NEX-GDDP-CMIP6) pr, convert it to apcp, and perform a quantile-mapping bias correction against NARR’s daily apcp (regridded via nearest neighbor to 0.25°) over 1979-2014. For each day and each 0.25° grid cell with CAPE or apcp above zero, the model generates a distribution of plausible flashrate from which we randomly sample a single daily realization. Finally, we bias-correct the resulting daily flashrate via quantile mapping, referencing the historical period (1987-2014) to ensure coherence with observed lightning data.

##### CAPE calculation for future scenarios

We calculate daily CAPE at 0.25° resolution by simulating the adiabatic ascent of a near-surface air parcel from 1000 hPa to 100 hPa in 100 hPa intervals. Let *i* = 1, …, 10 index these discrete pressure levels, where *i* = 1 corresponds to 1000 hPa and *i* = 10 to 100 hPa. CAPE represents the integrated energy a buoyant parcel can gain relative to its environment, making it a key measure of convective intensity and lightning potential under future climate conditions. Mathematically, CAPE is given by:

9 $$CAPE\,=\,\mathop{\sum }\limits_{i=1}^{10}\Delta P\,H({b}_{i})\,{b}_{i},$$

where Δ*P* = 100 hPa, *H*(⋅) is a Heaviside step function (zero if *b*_*i*_ ≤ 0), and

10 $${b}_{i}\,=\,\frac{1}{{\rho }_{p,i}}\,-\,\frac{1}{{\rho }_{e,i}},$$

the buoyancy term at level *i*. Here, *ρ*_*p*,*i*_ is the parcel density and *ρ*_*e*,*i*_ is the environmental density at level *i*. Positive *b*_*i*_ indicates the parcel is lighter than its surroundings, thus contributing to CAPE.

To evaluate *ρ*_*p*,*i*_ and *ρ*_*e*,*i*_ at each level *i*, we compute the parcel’s virtual temperature *T*_*v*,*i*_ = *T*_*i*_[1 + 0.61 *q*_*i*_] from the parcel temperature (*T*_*i*_) and specific humidity (*q*_*i*_) at level *i*, and obtain the environment’s temperature *T*_env,*i*_ from the temperature profile data described earlier. We then have:

11 $${\rho }_{p,i}\,=\,\frac{{P}_{i}}{{R}_{d}\,{T}_{v,i}},\quad \quad {\rho }_{e,i}\,=\,\frac{{P}_{i}}{{R}_{d}\,{T}_{env,i}},$$

where *P*_*i*_ is the pressure at level *i* and *R*_*d*_ ≈ 287 J kg^−1^ K^−1^ is the gas constant for dry air.

While the environmental density *ρ*_*e*,*i*_ can be directly calculated from the environment’s temperature *T*_env,*i*_, a central task for obtaining the parcel density *ρ*_*p*,*i*_ is to determine (*T*_*i*_,  *q*_*i*_) at each level *i*. We assume a reversible moist process in which the parcel’s equivalent potential temperature *θ*_*e*_ remains constant. Specifically,

12 $${\theta }_{e}(T,P,q)\,=\,T\,{(\frac{1000}{P})}^{\frac{{R}_{d}}{{C}_{p}}}\exp (\frac{{L}_{v}\,r}{{C}_{p}\,T}),\quad \quad r\,=\,\frac{q}{1-q},$$

where *T* is the parcel temperature (K), *P* is pressure (hPa), *r* is the mixing ratio, *q* is specific humidity, *C*_*p*_ ≈ 1004 J kg^−1^ K^−1^ is the specific heat at constant pressure, and *L*_*v*_ ≈ 2.5 × 10^6^ J kg^−1^ is the latent heat of vaporization. Near the surface, we compute

13 $${\theta }_{e,ns}={\theta }_{e}({T}_{ns},{P}_{ns},{q}_{ns}).$$

During ascent:

- **Below** the lifting condensation level, the parcel cools at the dry adiabatic lapse rate, maintaining *q* = *q*_ns_.
- **Above** saturation, it follows a moist-adiabatic lapse rate, updating *q* to local saturation at each 100 hPa step (e.g., via August-Roche-Magnus).

At each level *i*, we iteratively solve *θ*_*e*_(*T*_*i*_, *P*_*i*_, *q*_*i*_) = *θ*_*e*,ns_ using a root-finding method (e.g., Newton-Raphson), and obtain (*T*_*i*_,  *q*_*i*_). We then compute *T*_*v*,*i*_ and *ρ*_*p*,*i*_. Repeating this procedure up to 100 hPa provides the “parcel path.” Summing the positive buoyancy contributions at each step yields a daily CAPE value (in J ⋅ kg^−1^) for each 0.25° grid cell, which is then bias-corrected against historical data (1979-2014) using quantile-mapping to align with observed climatology.

### Wildfire (0.25°, daily)

We produce a daily wildfire dataset at 0.25° resolution spanning both historical (1984-2023) and future (through 2100) periods. The dataset provides three fields for each day and grid cell: (a) a binary indicator of whether there is a fire actively burning (fburn), (b) the fraction of population affected (fpop), and (c) the fraction of power transmission lines affected (fline). Below is a description of how these fields are derived for the historical period using observed fire-occurrence and perimeter data and for future periods using proxy relationships with fire weather indices from CMIP6 outputs.

#### Historical period (1984-2023)

To characterize historical wildfire incidents in the United States at 0.25° resolution, we merge two types of fire information:

- Occurrence, including discovery/containment dates and a unique fire ID.
- Perimeters, providing geospatial footprints of burned areas.

We unify multiple source datasets, including InFORM^13^, the Fire Program Analysis fire-occurrence database (FPA-FOD)^14^, WFIGS^15^, InterAgencyFirePerimeterHistory^16^, and GeoMAC^17^, removing apparent duplicates by cross-checking overlapping incidents with matching or near-matching ignition dates, fire names, or bounding polygons. Each occurrence record supplies discovery and containment dates (or controlled/out dates, if containment is unavailable). We say a fire is “active” on any calendar day *t* from its discovery date to its containment date (inclusive). The perimeter component delineates each fire’s burned area.

A daily 0.25° burning indicator is created by overlaying these fire perimeters on the 0.25° grid. A grid cell is marked as burning (fburn = 1) on day *t* if it intersects any active fire perimeter for that day; otherwise, the indicator is 0. Next, we estimate fpop in each grid cell *c* on day *t*. We overlay census block groups (BGs), each with known population, on the same 0.25° grid and check which block-group polygons intersect both cell *c* and at least one active fire perimeter on day *t*. We sum the affected population in those blocks and divide by the total population of all blocks intersecting cell *c*. Formally,

14 $$\,Fraction\,of\,population\,affected\,(c,t)\,=\,\frac{{\sum }_{\begin{array}{c}BG\cap c,BG\cap FirePerim(t)\ne {\rm{\varnothing }}\end{array}}Pop(BG)}{{\sum }_{BG\cap c}Pop(BG)}.$$

Similarly, we estimate fline by overlaying a vector dataset of transmission lines^72^ and noting which lines (or segments) pass through a burning area on day *t* in cell *c*. We then compute

15 $$\,Fraction\,of\,lines\,affected\,(c,t)\,=\,\frac{\#\{Lines\cap c\,:Line\cap FirePerim(t)\ne {\rm{\varnothing }}\}}{\#\{Lines\cap c\}},$$

where the numerator is the count of lines intersecting active fires on day *t*, and the denominator is the total lines intersecting cell *c*. Collectively, these three daily fields—fburn, fpop, and fline—form our historical wildfire dataset at 0.25° resolution.

#### Future projections (through 2100)

Because CMIP6 GCMs (and their downscaled products) typically lack direct wildfire outputs, we use an indirect method to estimate the same three fields for future scenarios. First, we estimate daily burning probability at 0.25°, then obtain fburn by stochastically determining whether a given cell ignites on a particular day. Next, we infer fpop and fline using a Fire Weather Index (FWI)-based analog to historical fires.

Daily burning probability is linked to the Fine Fuel Moisture Code (FFMC), which ranges from 0 to 101 and signifies surface-fuel flammability. We parameterize burn probability to rise gradually with FFMC until 65 and more steeply beyond 65^73,74^

16 $$BurnProbability\,=\,K\,(0.\,1\,\min (FFMC,65)\,+\,\exp (0.\,15\,{[FFMC-65]}^{+})),$$

where ${[FFMC-65]}^{+}=\max (FFMC\,-\,65,\,0)$ and *K* is a cell-specific scaling constant. We derive *K* so that the average burn probability from this formula between 2003 and 2023 (wildfire data quality and completeness in earlier years are limited due to less advanced wildfire monitoring) matches the empirical burn probability, inferred from the observed number of burning days in each 0.25° cell over the same time period. If a cell’s historical fire data appear underreported, we cross-check with an alternate *K* estimated from published burn probability maps^75^, ultimately selecting the larger estimate. To facilitate these estimates of K and later bias correction for future values, we obtain historical FFMC data from ERA5 at 0.25° for 2003-2023 and apply it to each cell’s burn-probability calculation based on equation (16).

Future monthly FFMC values at 0.25° for each of the 14 GCMs (available via NEX-GDDP-FWI^76^) are bias-corrected against ERA5 (1984-2014) using variance scaling. We assign this monthly FFMC value to every day in the month, then compute daily burn probabilities at 0.25° based on equation (16). A random draw from this probability determines whether fburn is 1 or 0 on a given day. This approach does not track multi-day fires explicitly; rather, it treats each day independently so that the total burning days match the overall probability distribution.

Once a day is flagged as burning, we estimate fpop and fline using FWI, which correlates positively with fire intensity and spatial extent^74^. After bias-correcting monthly FWI at 0.25° from the same 14 GCMs (also available via NEX-GDDP-FWI), we identify, for each burning day in each cell, the historical burning day (1984-2023) in that cell whose FWI is closest to the future day’s FWI. We then assign the historical day’s fpop/fline to the future day. To facilitate the bias correction and the analog day search, we obtain historical FWI data from ERA5 at 0.25°. Because historical records capture observed variability in fire extent, this analog approach preserves realistic spatial patterns of fire impacts while allowing us to project changes in burning probability under altered climate conditions.

The resulting future projection provides daily 0.25° fields of fburn, fpop, and fline for each of the 14 CMIP6 models, under the historical simulation, SSP245, and SSP585.

## Data Records

All data generated in this study is publicly available at the Stanford Digital Repository (10.25740/xz676qm0434) and spans the period 1979-2100 at 0.25° resolution across the United States^77^. Table 1 summarizes the included variables, each provided at daily or sub-daily resolution in netCDF-4 files. Within the repository, historical files are stored in a top-level folder named historical, with subfolders matching each variable name (e.g., historical/fg10/). Future projection files reside in the cmip6 folder, likewise organized by variable (e.g., cmip6/fg10/).

For historical data (1979-2023), sensor-based observations and reanalysis outputs are unified into files for each year named according to:


{variable}_{frequency}_{year}.nc


For example, fg10_6hr_1990.nc contains 6-hourly maximum 10m wind gusts for 1990. Each file holds a single variable on dimensions (lat, lon, time), or (lat, lon, time, level) if vertical profiles (e.g., temperature on pressure levels) are included.

Future projections draw on 14 CMIP6 models under three experiments: historical (1979-2014), SSP245 (2015-2100), and SSP585 (2015-2100). These files use filenames of the form:


{variable}_{frequency}_{model}_{experiment}_{variant}_{grid_label}_{year}.nc


Here, *model* is the GCM name (e.g., CNRM-CM6-1), *experiment* is one of historical, ssp245, or ssp585, *variant* denotes the realization (e.g., r1i1p1f1), and *grid_label* indicates the original model’s labeling scheme (e.g., gn, gr). For instance, fg10_6hr_CNRM-CM6-1_ssp585_r1i1p1f2_gr_2060.nc designates 6-hourly maximum 10 m wind gusts from CNRM-CM6-1 under SSP585 in 2060. Like the historical data, each file covers a single calendar year and contains one variable at daily or sub-daily resolution. This structure supports selective downloading by variable, time range, or GCM, facilitating comprehensive analyses of infrastructure-critical weather and climate hazards.

## Technical Validation

From the previous sections, our dataset of infrastructure-critical weather and climate variables comprises (a) a historical dataset (HD) calibrated from observations and reanalysis, and (b) derived fields from 14 CMIP6 GCMs under their historical simulation (HS), SSP245, and SSP585 experiments. As one illustrative example of how these derived variables compare to observations and how they might evolve in the future, we examine MRI-ESM2-0 (one of the 14 GCMs) by comparing its HS with HD, analyzing both a mid-of-century (MoC, 2045-2059) period and long-term (2015-2100) nationwide trends in mean and extreme conditions under SSP585. Other GCMs and experiments in our dataset may differ from this regionally or at national scale, so the following patterns and trends should be viewed as one possible case of a future climate simulation.

**Fig. 1:**
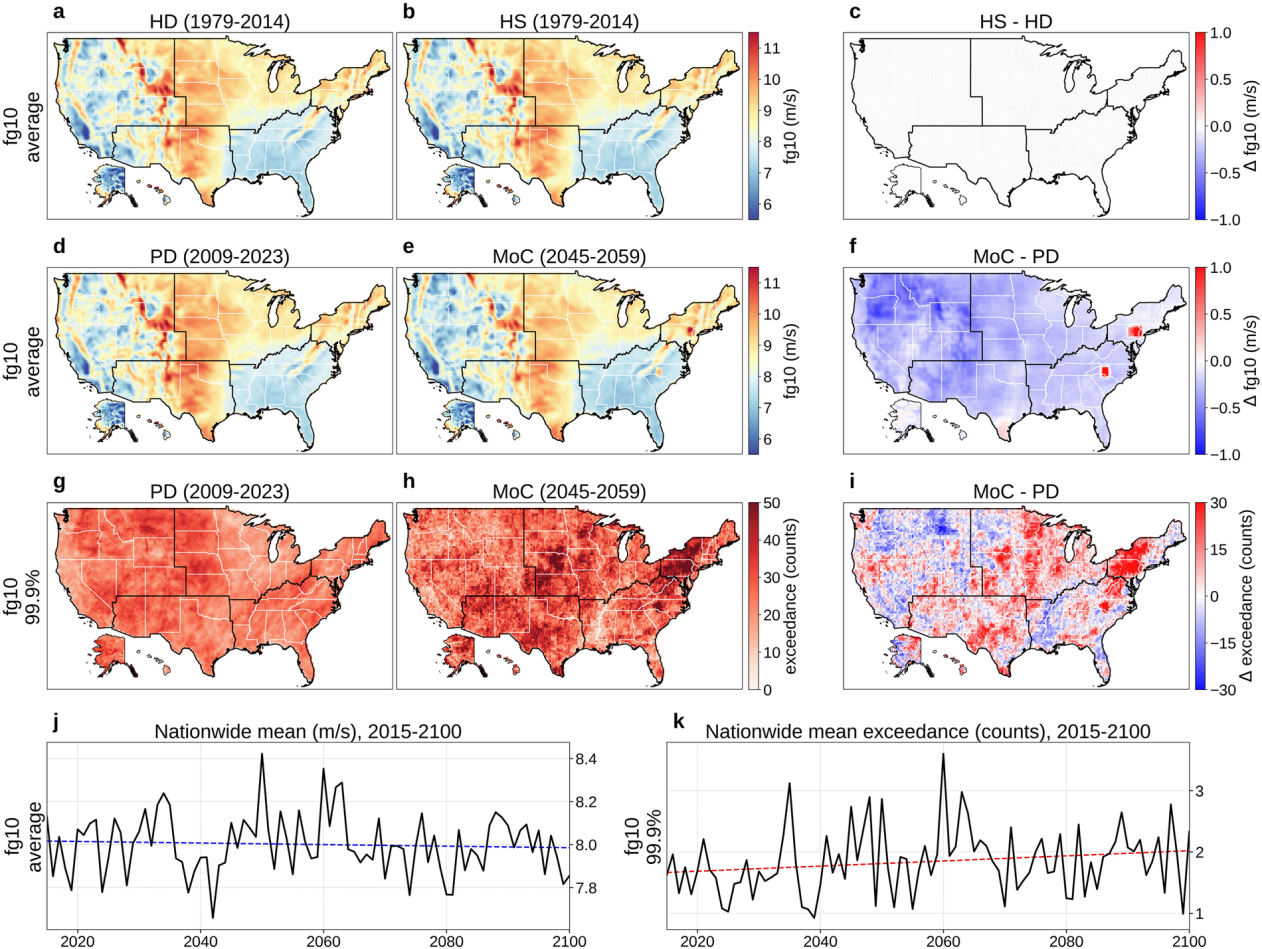
Overview of 6-hourly maximum 10 m wind gust (fg10). (**a**) Mean 6-hourly fg10 (1979-2014) from the historical dataset (HD). (**b**) Mean 6-hourly fg10 (1979-2014) from a historical simulation (HS). (**c**) HS  − HD (1979-2014). (**d**) Mean 6-hourly fg10 for a present-day (PD) period (2009-2023) of HD. (**e**) Mean 6-hourly fg10 for a mid-of-century (MoC) period (2045-2059). (**f**) MoC  − PD for mean 6-hourly fg10. (**g**) Absolute frequency of 6-hourly fg10 above the 99.9th percentile (reference period: 1979-2023) during PD. (**h**) Same frequency during MoC. (**i**) MoC  − PD for that extreme frequency. (**j**) Nationwide population-weighted average of mean 6-hourly fg10, 2015-2100. (**k**) Nationwide population-weighted average absolute frequency of 6-hourly fg10 above the 99.9th percentile (reference period: 1979-2023), 2015-2100. Trend lines in panels (**j,k**) are colored red for increasing trends and blue for decreasing. Panels (**b,e,h,j,k**) show results from MRI-ESM2-0, one of the 14 CMIP6 GCMs in our dataset, with (**e,h,j,k**) evaluated under SSP585.

Figure 1a,b present the 36-year (1979-2014) mean of 6-hourly fg10 at 0.25° for HD and HS, respectively, while Fig. 1c shows their difference (HS  − HD). The bias correction performs as intended, with minimal differences evident in Fig. 1c. To quantitatively assess distributional alignment, we computed the average Kullback-Leibler (KL) divergence^78^ (0.004) and Jensen-Shannon (JS) distance^79^ (0.027) between HD and HS empirical probability distribution functions (PDFs) at the 6-hourly level across all grid cells, where lower values indicate better alignment. The slight distributional differences arise from the discrete quantile bins used in the bias correction procedure. We define a present-day (PD) period (2009-2023) within HD and compare its mean 6-hourly fg10 to that of the MoC period in Fig. 1d,e, with Fig. 1f illustrating MoC  − PD. Fig. 1f indicates a general decrease in mean gust speed across much of the United States as opposed to the alignment shown in Fig. 1c. The average KL divergence (0.016) and JS distance (0.051) between PD and MoC PDFs are higher than those between HD and HS. Figure 1g,h map the absolute frequency of 6-hourly fg10 exceeding the 99.9th percentile (referencing 1979-2023) for PD and MoC, respectively, and Fig. 1i shows MoC  − PD for that extreme frequency. Over the long term (2015-2100), Fig. 1j,k present the nationwide population-weighted average of mean and extreme 6-hourly fg10. These long-term trajectories are subject to substantial year-to-year variability and should be interpreted as broad trends rather than strictly monotonic changes.

**Fig. 2:**
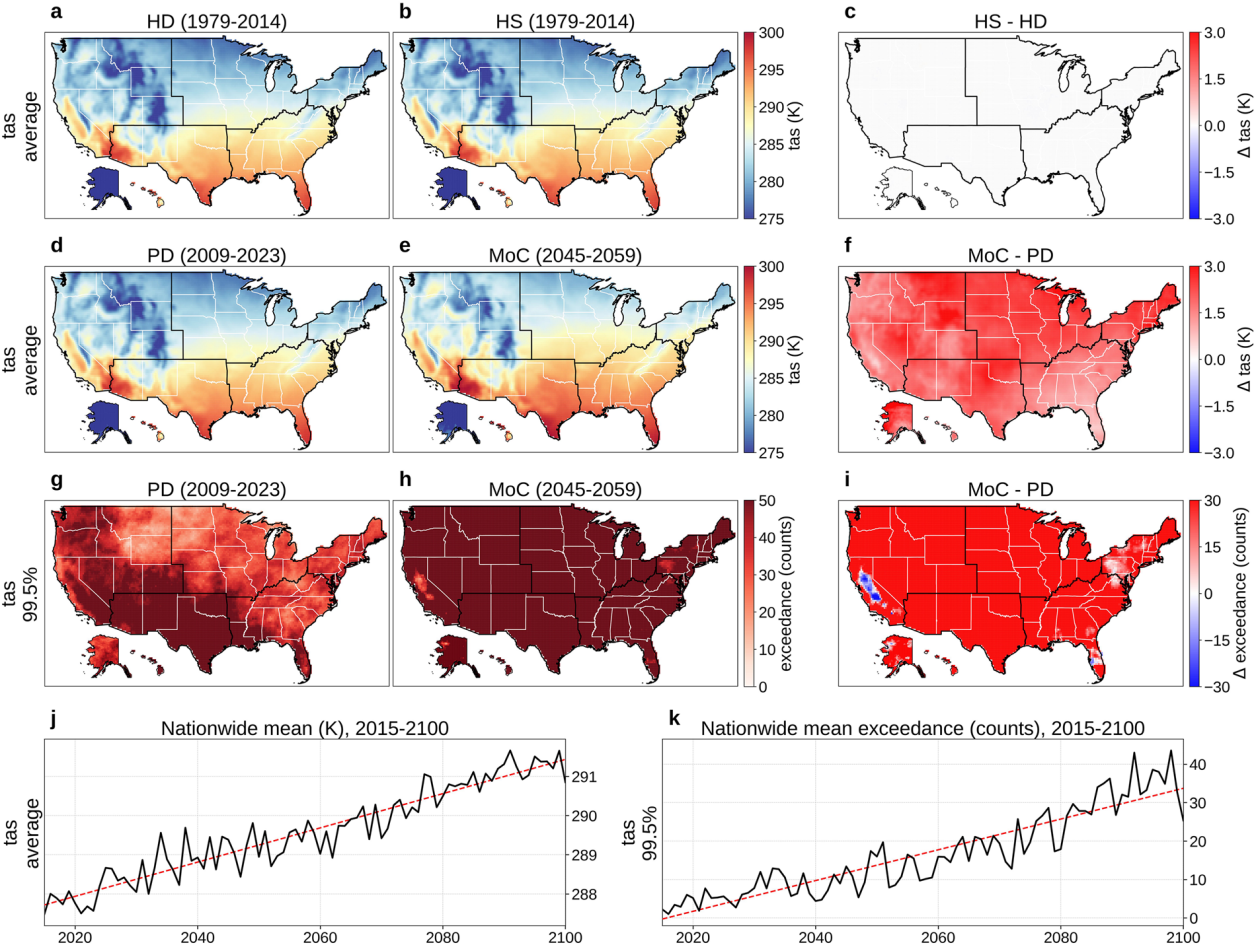
Overview of daily near-surface air temperature (tas). (**a**) Mean daily tas (1979-2014) from the historical dataset (HD). (**b**) Mean daily tas (1979-2014) from a historical simulation (HS). (**c**) HS  − HD (1979-2014). (**d**) Mean daily tas for a present-day (PD) period (2009-2023) of HD. (**e**) Mean daily tas for a mid-of-century (MoC) period (2045-2059). (**f**) MoC  − PD for mean daily tas. (**g**) Absolute frequency of daily tas above the 99.5th percentile (reference period: 1979-2023) during PD. (**h**) Same frequency during MoC. (**i**) MoC  − PD for that extreme frequency. (**j**) Nationwide population-weighted average of mean daily tas, 2015-2100. (**k**) Nationwide population-weighted average absolute frequency of daily tas above the 99.5th percentile (reference period: 1979-2023), 2015-2100. Trend lines in panels (**j,k**) are colored red for increasing trends and blue for decreasing. Panels (**b,e,h,j,k**) show results from MRI-ESM2-0, one of the 14 CMIP6 GCMs in our dataset, with (**e,h,j,k**) evaluated under SSP585.

**Fig. 3:**
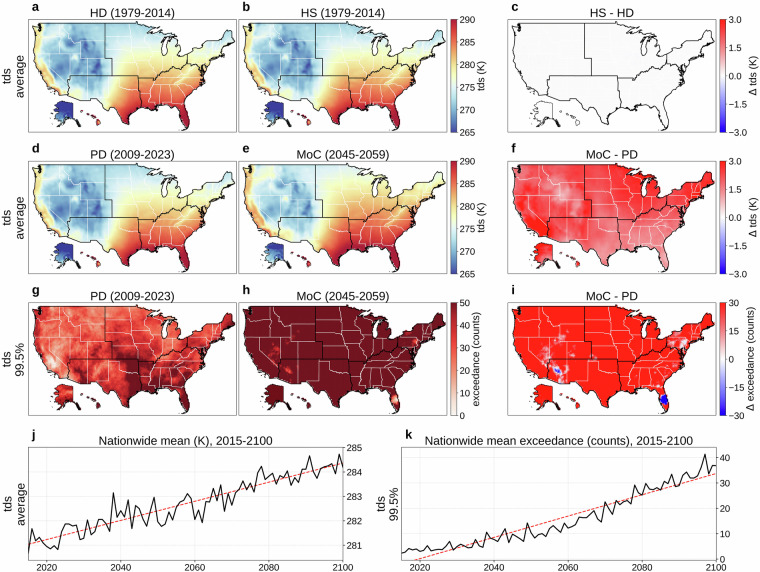
Overview of daily near-surface dew point temperature (tds). (**a**) Mean daily tds (1979-2014) from the historical dataset (HD). (**b**) Mean daily tds (1979-2014) from a historical simulation (HS). (**c**) HS  − HD (1979-2014). (**d**) Mean daily tds for a present-day (PD) period (2009-2023) of HD. (**e**) Mean daily tds for a mid-of-century (MoC) period (2045-2059). (**f**) MoC  − PD for mean daily tds. (**g**) Absolute frequency of daily tds above the 99.5th percentile (reference period: 1979-2023) during PD. (**h**) Same frequency during MoC. (**i**) MoC  − PD for that extreme frequency. (**j**) Nationwide population-weighted average of mean daily tds, 2015-2100. (**k**) Nationwide population-weighted average absolute frequency of daily tds above the 99.5th percentile (reference period: 1979-2023), 2015-2100. Trend lines in panels (**j,k**) are colored red for increasing trends and blue for decreasing. Panels (**b,e,h,j,k**) show results from MRI-ESM2-0, one of the 14 CMIP6 GCMs in our dataset, with (**e,h,j,k**) evaluated under SSP585.

**Fig. 4:**
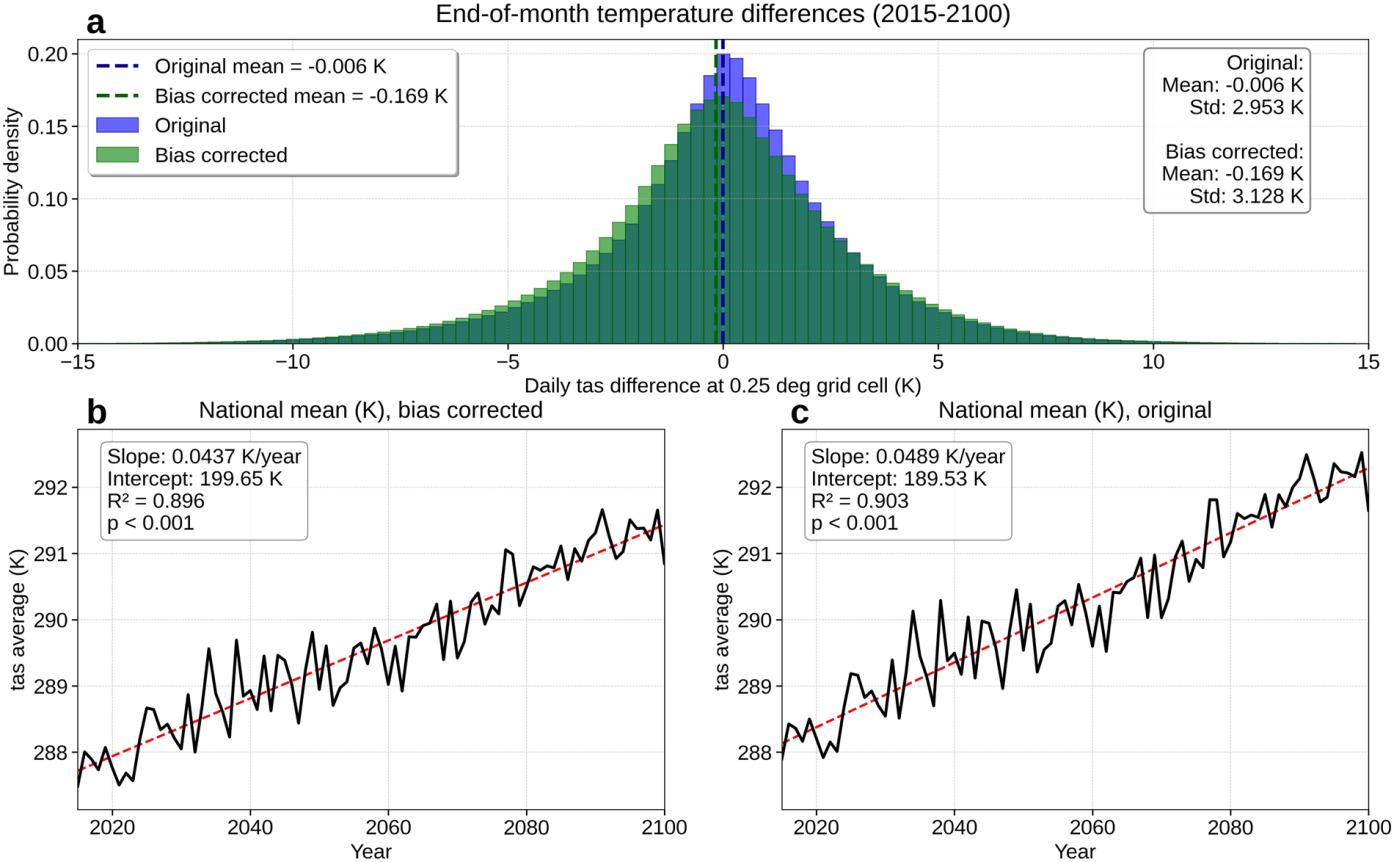
Validation of bias correction methodology for near-surface air temperature (tas). (**a**) Probability density distributions of tas differences between the last day of each month and the first day of the following month for all grid cells (2015-2100, SSP585) comparing our bias-corrected output (mean -0.169 K, standard deviation 3.128 K) with original NEX-GDDP-CMIP6 (mean -0.006 K, standard deviation 2.953 K). (**b**) U.S. nationwide mean tas trend under SSP585 (2015-2100) after our bias correction showing warming trend of 0.0437 K/year. (**c**) Original NEX-GDDP-CMIP6 U.S. nationwide mean tas trend under SSP585 (2015-2100) showing warming trend of 0.0489 K/year. All panels show results from MRI-ESM2-0.

Figures 2 and 3 respectively present daily tas and tds. Panels (a) and (b) show the 36-year (1979-2014) mean for HD and HS, and (c) maps HS  − HD; in both variables, these comparisons reveal negligible differences between the HD and HS (average KL divergence: 0.010 for tas, 0.010 for tds; JS distance: 0.037 for tas, 0.039 for tds). These low values demonstrate the effectiveness of our additional bias correction; before correction, NEX-GDDP-CMIP6 temperature showed larger differences from ERA5 observations (KL divergence: 0.094; JS distance: 0.105). Our monthly quantile mapping approach for bias correction introduces only minor discontinuities at month boundaries, with tas differences between consecutive months showing closely aligned distributions to the original data (our bias-corrected output: mean −0.169 K, standard deviation 3.128 K; original NEX-GDDP-CMIP6: mean −0.006 K, standard deviation 2.953 K; Fig. 4a). Panels (d) and (e) compare PD and MoC means, with (f) showing MoC  − PD; across nearly the entire United States, mean tas and tds increase in contrast to what is shown in panel (c) (average KL divergence: 0.164 for tas, 0.135 for tds; JS distance: 0.156 for tas, 0.152 for tds). Panels (g) and (h) map the absolute frequency of daily tas or tds exceeding the 99.5th percentile (referencing 1979-2023) for PD and MoC, respectively, and (i) illustrates MoC  − PD for these extremes. Over the long term (2015-2100), panels (j) and (k) present the nationwide population-weighted average of mean and extreme tas or tds, which steadily rise with relatively small year-to-year variability compared to other variables. Our bias correction does not include the detrending-retrending method used in the original NEX-GDDP-CMIP6 development, thus we note here the difference between long-term tas trends of the two: our result's warming trend in Fig. 4b (0.0437 K/year) has a lower slope than the original NEX-GDDP-CMIP6 trend in Fig. 4c (0.0489 K/year).

**Fig. 5:**
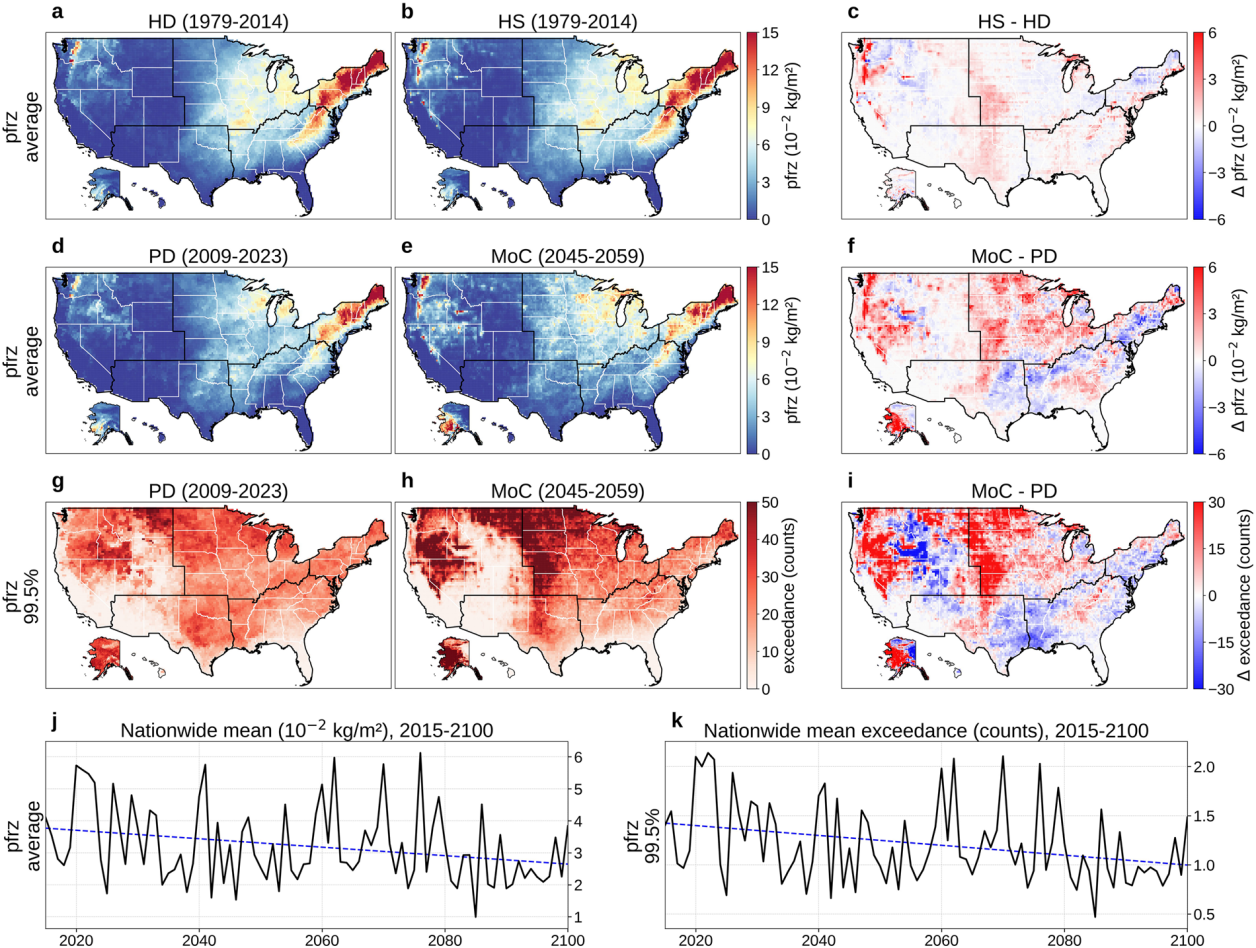
Overview of daily freezing-rain or ice-pellet (pfrz). (**a**) Mean daily pfrz (1979-2014) from the historical dataset (HD). (**b**) Mean daily pfrz (1979-2014) from a historical simulation (HS). (**c**) HS  − HD (1979-2014). (**d**) Mean daily pfrz for a present-day (PD) period (2009-2023) of HD. (**e**) Mean daily pfrz for a mid-of-century (MoC) period (2045-2059). (**f**) MoC  − PD for mean daily pfrz. (**g**) Absolute frequency of daily pfrz above the 99.5th percentile (reference period: 1979-2023) during PD. (**h**) Same frequency during MoC. (**i**) MoC  − PD for that extreme frequency. (**j**) Nationwide population-weighted average of mean daily pfrz, 2015-2100. (**k**) Nationwide population-weighted average absolute frequency of daily pfrz above the 99.5th percentile (reference period: 1979-2023), 2015-2100. Trend lines in panels (**j,k**) are colored red for increasing trends and blue for decreasing. Panels (**b,e,h,j,k**) show results from MRI-ESM2-0, one of the 14 CMIP6 GCMs in our dataset, with (**e,h,j,k**) evaluated under SSP585.

**Fig. 6:**
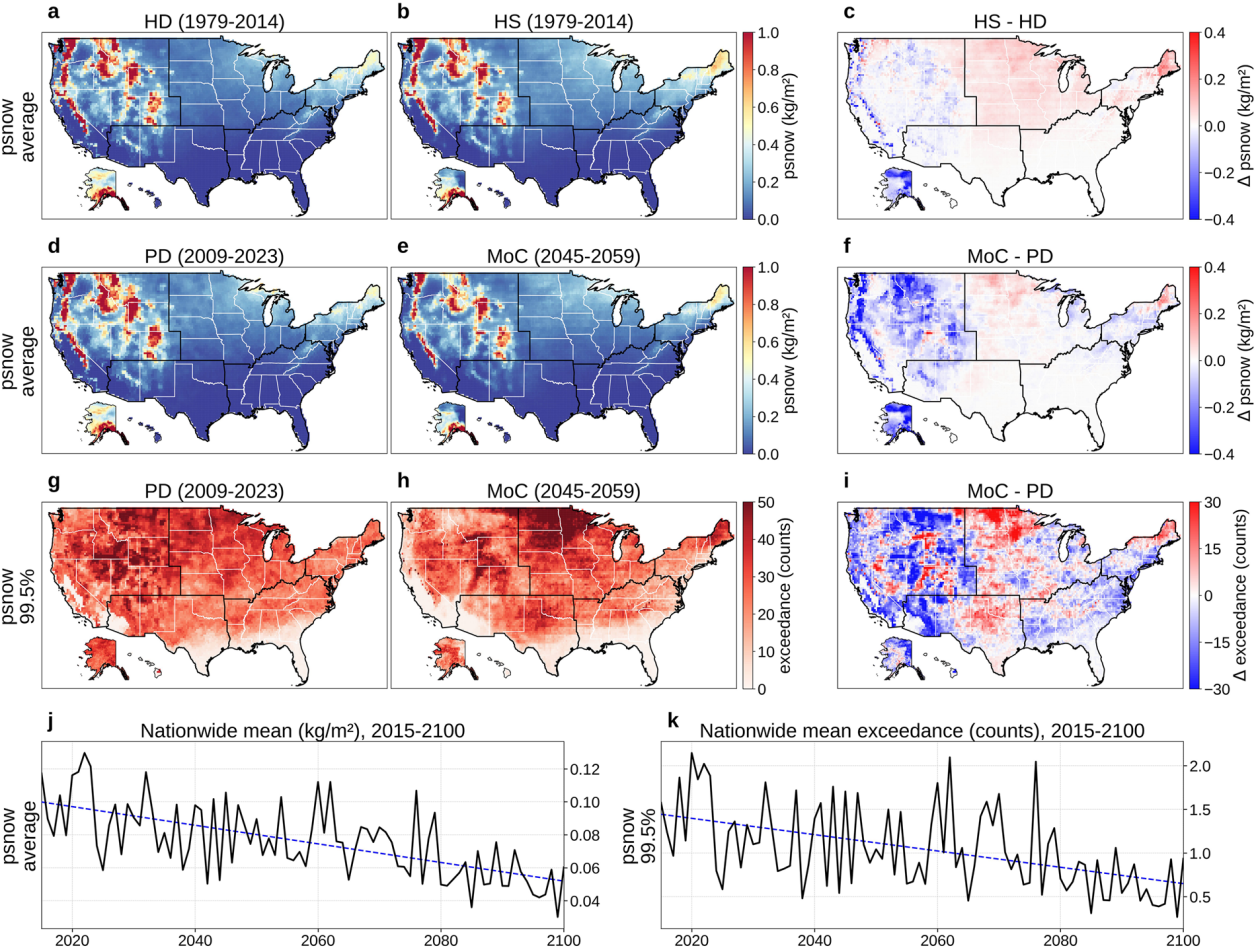
Overview of daily snowfall (psnow). (**a**) Mean daily psnow (1979-2014) from the historical dataset (HD). (**b**) Mean daily psnow (1979-2014) from a historical simulation (HS). (**c**) HS  − HD (1979-2014). (**d**) Mean daily psnow for a present-day (PD) period (2009-2023) of HD. (**e**) Mean daily psnow for a mid-of-century (MoC) period (2045-2059). (**f**) MoC  − PD for mean daily psnow. (**g**) Absolute frequency of daily psnow above the 99.5th percentile (reference period: 1979-2023) during PD. (**h**) Same frequency during MoC. (**i**) MoC  − PD for that extreme frequency. (**j**) Nationwide population-weighted average of mean daily psnow, 2015-2100. (**k**) Nationwide population-weighted average absolute frequency of daily psnow above the 99.5th percentile (reference period: 1979-2023), 2015-2100. Trend lines in panels (**j,k**) are colored red for increasing trends and blue for decreasing. Panels (**b,e,h,j,k**) show results from MRI-ESM2-0, one of the 14 CMIP6 GCMs in our dataset, with (**e,h,j,k**) evaluated under SSP585.

**Fig. 7:**
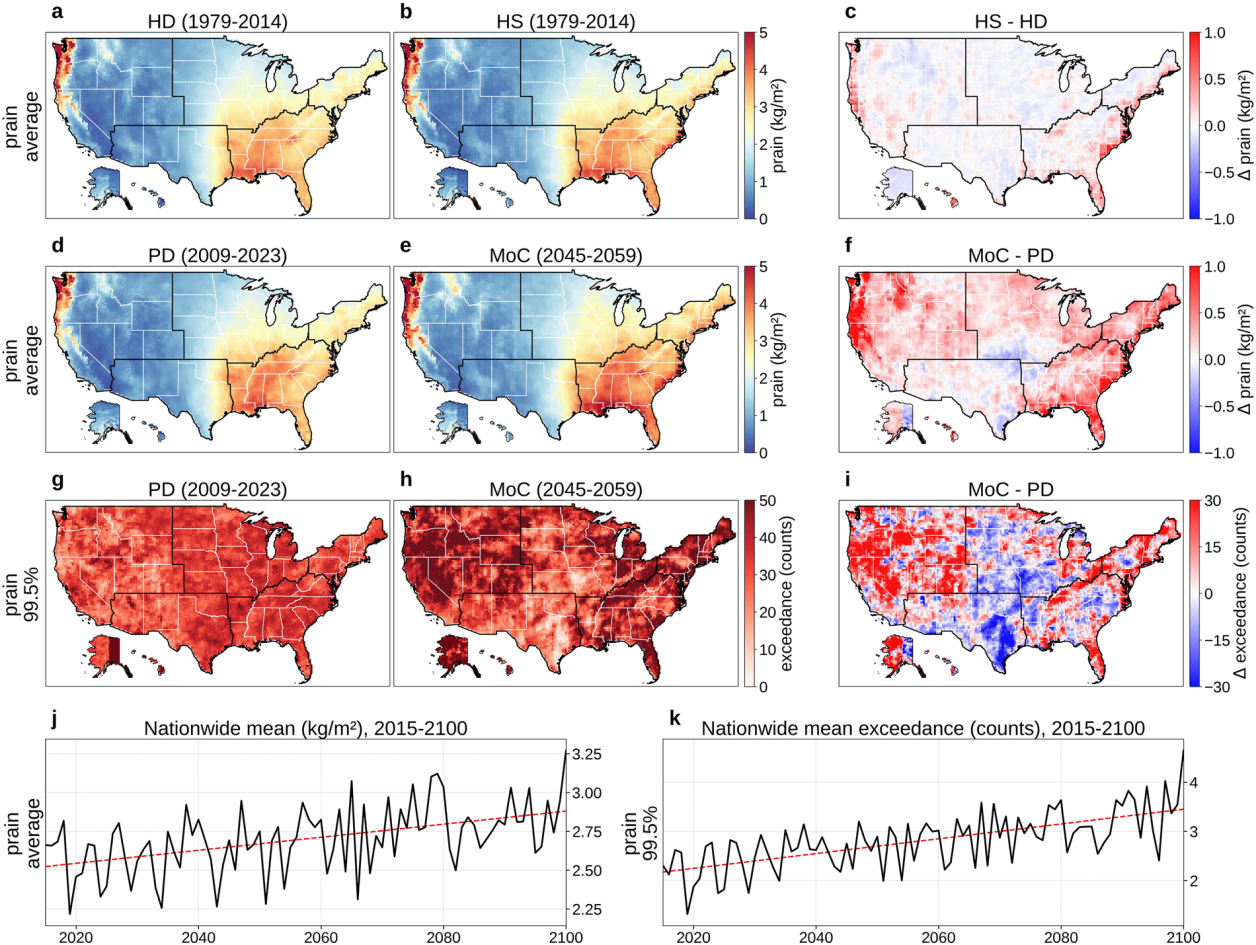
Overview of daily rainfall (prain). (**a**) Mean daily prain (1979-2014) from the historical dataset (HD). (**b**) Mean daily prain (1979-2014) from a historical simulation (HS). (**c**) HS  − HD (1979-2014). (**d**) Mean daily prain for a present-day (PD) period (2009-2023) of HD. (**e**) Mean daily prain for a mid-of-century (MoC) period (2045-2059). (**f**) MoC  − PD for mean daily prain. (**g**) Absolute frequency of daily prain above the 99.5th percentile (reference period: 1979-2023) during PD. (**h**) Same frequency during MoC. (**i**) MoC  − PD for that extreme frequency. (**j**) Nationwide population-weighted average of mean daily prain, 2015-2100. (**k**) Nationwide population-weighted average absolute frequency of daily prain above the 99.5th percentile (reference period: 1979-2023), 2015-2100. Trend lines in panels (**j,k**) are colored red for increasing trends and blue for decreasing. Panels (**b,e,h,j,k**) show results from MRI-ESM2-0, one of the 14 CMIP6 GCMs in our dataset, with (**e,h,j,k**) evaluated under SSP585.

Figures 5, 6, and 7 respectively present daily pfrz, psnow, and prain. Panels (a) and (b) show the 36-year (1979-2014) mean for HD and HS, with (c) mapping HS  − HD. Although the overall alignment between HS and HD is good, each variable exhibits some regional mismatches (average KL divergence: 0.019 for pfrz, 0.067 for psnow, 0.051 for prain; JS distance: 0.035 for pfrz, 0.098 for psnow, 0.077 for prain), stemming from model biases, our derivation of temperature profiles, the precipitation-type classification, and our bias correction that focuses on distributions rather than occurrence frequency. Panels (d) and (e) compare PD and MoC means, with (f) showing MoC  − PD (average KL divergence: 0.046 for pfrz, 0.082 for psnow, 0.086 for prain; JS distance: 0.046 for pfrz, 0.107 for psnow, 0.097 for prain). Panels (g) and (h) depict the absolute frequency of daily pfrz, psnow, or prain exceeding the 99.5th percentile (referencing 1979-2023) during PD and MoC, and (i) shows MoC  − PD for these extremes. Finally, panels (j) and (k) present the nationwide population-weighted averages of mean and extreme precipitation over 2015-2100. Freezing rain (Fig. 5j,k) and snowfall (Fig. 6j,k) exhibit overall downward trends, whereas rainfall (Fig. 7j,k) shows a steady upward trajectory, aligning with the temperature trend in Fig. 2j,k. In all three cases, substantial year-to-year fluctuations mean these future paths should be viewed as broad patterns rather than uniform annual changes.

**Fig. 8:**
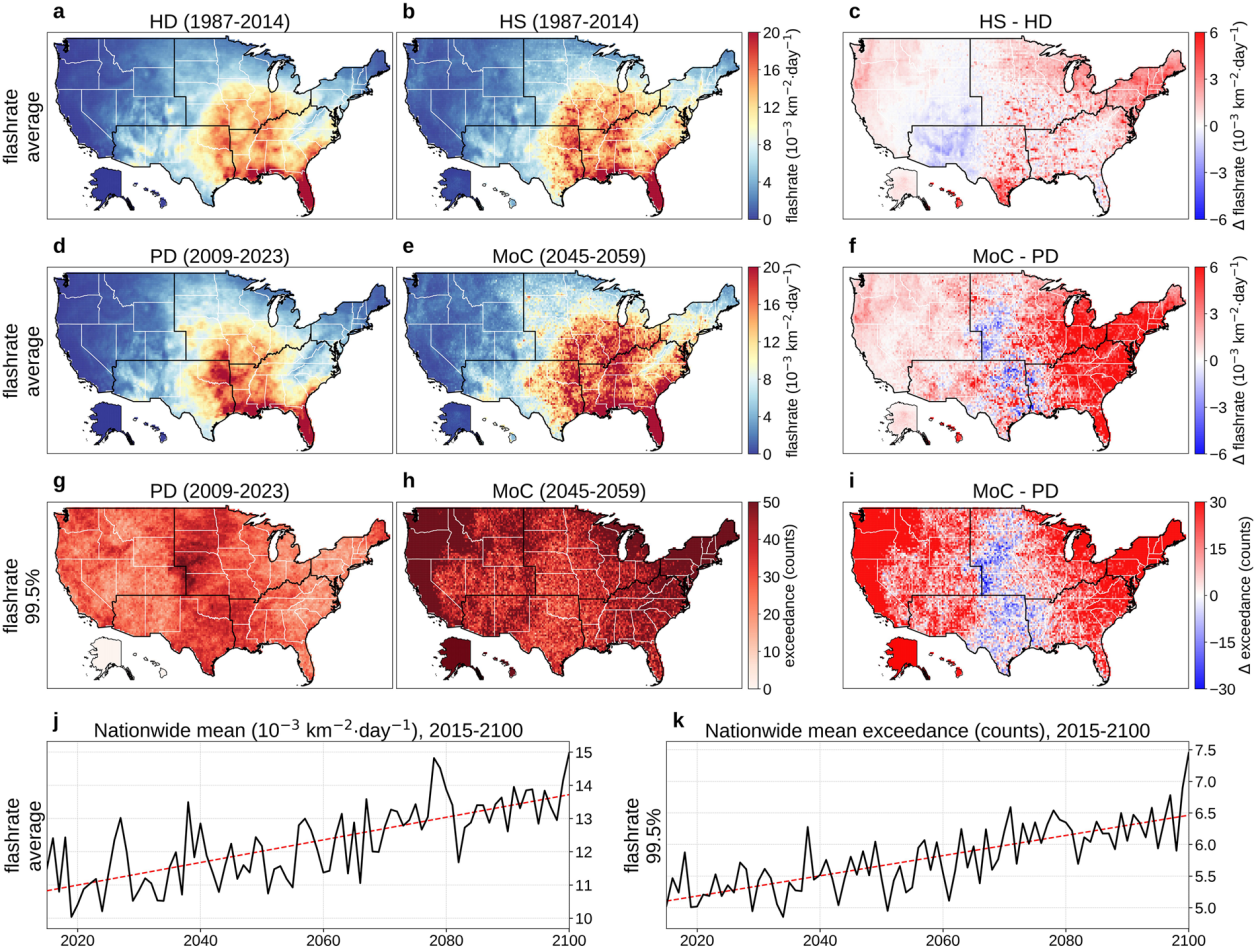
Overview of daily lightning flash rate (flashrate). (**a**) Mean daily flashrate (1987-2014) from the historical dataset (HD). (**b**) Mean daily flashrate (1987-2014) from a historical simulation (HS). (**c**) HS  − HD (1987-2014). (**d**) Mean daily flashrate for a present-day (PD) period (2009-2023) of HD. (**e**) Mean daily flashrate for a mid-of-century (MoC) period (2045-2059). (**f**) MoC  − PD for mean daily flashrate. (**g**) Absolute frequency of daily flashrate above the 99.5th percentile (reference period: 1987-2023) during PD. (**h**) Same frequency during MoC. (**i**) MoC  − PD for that extreme frequency. (**j**) Nationwide population-weighted average of mean daily flashrate, 2015-2100. (**k**) Nationwide population-weighted average absolute frequency of daily flashrate above the 99.5th percentile (reference period: 1987-2023), 2015-2100. Trend lines in panels (**j,k**) are colored red for increasing trends and blue for decreasing. Panels (**b,e,h,j,k**) show results from MRI-ESM2-0, one of the 14 CMIP6 GCMs in our dataset, with (**e,h,j,k**) evaluated under SSP585.

Figure 8a,b show the 28-year (1987-2014) mean daily flashrate at 0.25° for HD and HS, respectively, while Fig. 8c displays their difference (HS  − HD). Although minor misalignment appears between HS and HD—likely due to the stochastic nature of deriving flashrate from CAPE and precipitation–overall agreement remains satisfactory (average KL divergence: 0.296; JS distance: 0.091). Figure 8d,e compare PD (2009-2023) and MoC (2045-2059) means, with Fig. 8f illustrating MoC  − PD (average KL divergence: 0.612; JS distance: 0.106). Panels (g) and (h) map the absolute frequency of daily flashrate exceeding the 99.5th percentile (referencing 1987-2023) for PD and MoC, and Fig. 8i shows MoC  − PD for that extreme measure. Figure 8j,k present the nationwide population-weighted average of mean and extreme daily flashrate over 2015-2100, showing a steady upward trend, aligning with the trend of rainfall in Fig. 7j,k.

**Fig. 9:**
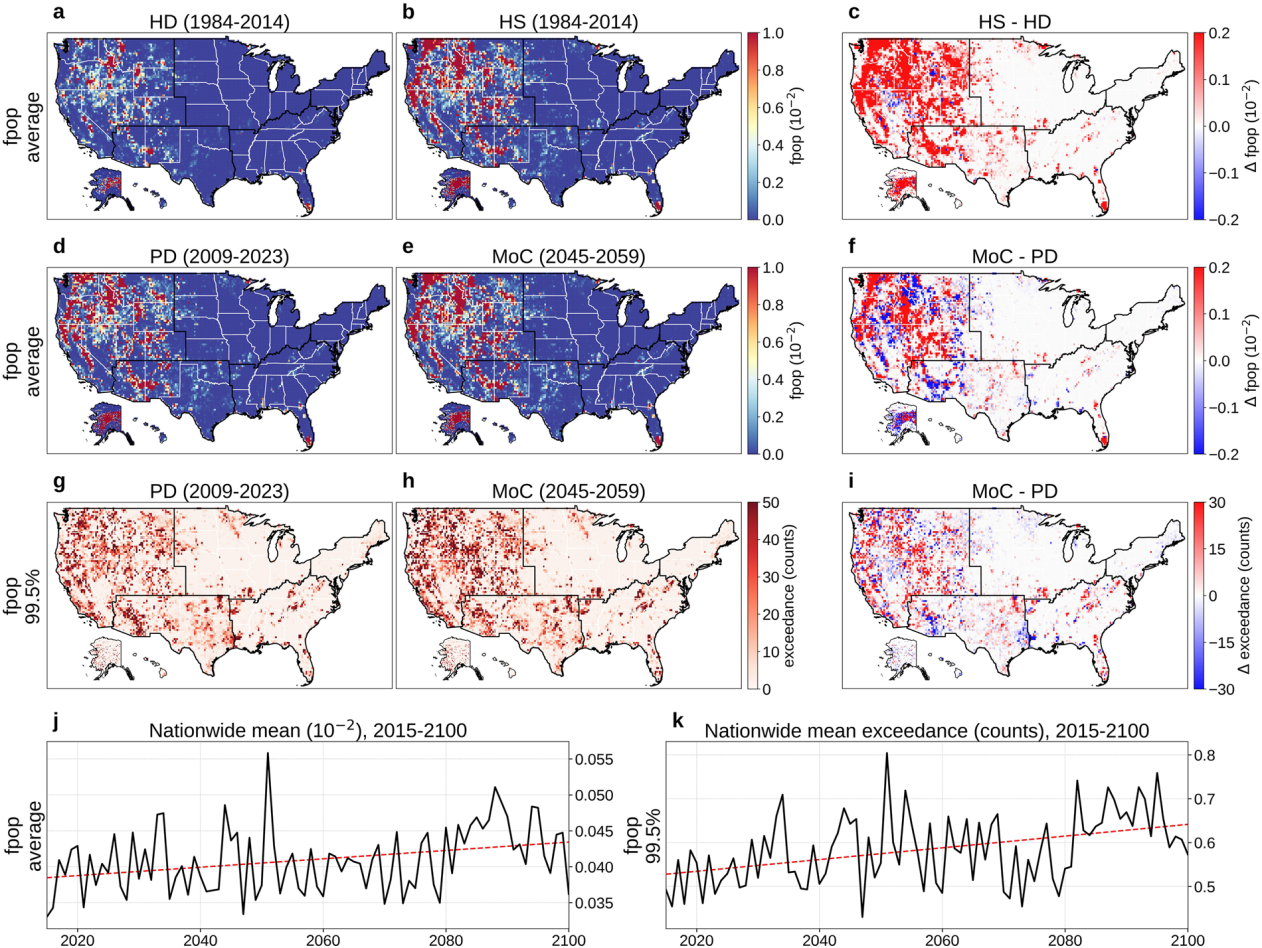
Overview of daily fraction of population affected by fires (fpop). (**a**) Mean daily fpop (1984-2014) from the historical dataset (HD). (**b**) Mean daily fpop (1984-2014) from a historical simulation (HS). (**c**) HS  − HD (1979-2014). (**d**) Mean daily fpop for a present-day (PD) period (2009-2023) of HD. (**e**) Mean daily fpop for a mid-of-century (MoC) period (2045-2059). (**f**) MoC  − PD for mean daily fpop. (**g**) Absolute frequency of daily fpop above the 99.5th percentile (reference period: 1984-2023) during PD. (**h**) Same frequency during MoC. (**i**) MoC  − PD for that extreme frequency. (**j**) Nationwide population-weighted average of mean daily fpop, 2015-2100. (**k**) Nationwide population-weighted average absolute frequency of daily fpop above the 99.5th percentile (reference period: 1984-2023), 2015-2100. Trend lines in panels (**j,k**) are colored red for increasing trends and blue for decreasing. Panels (**b,e,h,j,k**) show results from MRI-ESM2-0, one of the 14 CMIP6 GCMs in our dataset, with (**e,h,j,k**) evaluated under SSP585.

**Fig. 10:**
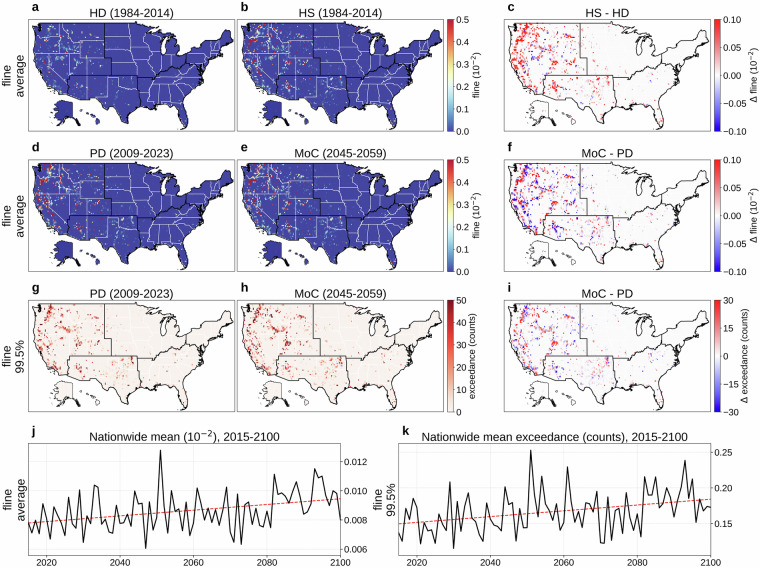
Overview of daily fraction of power transmission lines affected by fires (fline). (**a**) Mean daily fline (1984-2014) from the historical dataset (HD). (**b**) Mean daily fline (1984-2014) from a historical simulation (HS). (**c**) HS  − HD (1984-2014). (**d**) Mean daily fline for a present-day (PD) period (2009-2023) of HD. (**e**) Mean daily fline for a mid-of-century (MoC) period (2045-2059). (**f**) MoC  − PD for mean daily fline. (**g**) Absolute frequency of daily fline above the 99.5th percentile (reference period: 1984-2023) during PD. (**h**) Same frequency during MoC. (**i**) MoC  − PD for that extreme frequency. (**j**) Nationwide population-weighted average of mean daily fline, 2015-2100. (**k**) Nationwide population-weighted average absolute frequency of daily fline above the 99.5th percentile (reference period: 1984-2023), 2015-2100. Trend lines in panels (**j,k**) are colored red for increasing trends and blue for decreasing. Panels (**b,e,h,j,k**) show results from MRI-ESM2-0, one of the 14 CMIP6 GCMs in our dataset, with (**e,h,j,k**) evaluated under SSP585.

Figures 9 and 10 respectively present daily fpop and daily fline. Panels (a) and (b) show their mean values (1984-2014) from HD and HS, with (c) mapping HS  − HD. Unlike in other variables, evident mismatches between HS and HD appear due to incomplete wildfire historical records in earlier decades (average KL divergence: 0.030 for fpop, 0.003 for fline; JS distance: 0.017 for fpop, 0.002 for fline). Panels (d) and (e) compare PD (2009-2023) and MoC (2045-2059) means, with (f) illustrating MoC  − PD (average KL divergence: 0.012 for fpop, 0.002 for fline; JS distance: 0.013 for fpop, 0.002 for fline). Panels (g) and (h) depict the absolute frequency of daily fpop or fline exceeding the 99.5th percentile (reference period: 1984-2023) during PD and MoC, while (i) shows MoC  − PD for these extremes. Panels (j) and (k) present the nationwide population-weighted averages of mean and extreme fpop or fline over 2015-2100, each showing an overall upward trend despite high year-to-year variability.

## Usage Notes

An important use of this dataset is to facilitate robust assessments of how historical extreme weather events affect infrastructure systems in the United States, including but not limited to electric grids, natural gas pipelines, roads, rail networks, ports, and telecommunications. By providing wind gusts, temperature, dew point, precipitation (partitioned into rain, snow, and freezing rain or ice pellets), lightning, and wildfire metrics at 0.25° resolution with daily or sub-daily frequency, users can evaluate local-scale impacts that may trigger infrastructure failures. These data can be readily paired with additional event-based records—such as realized economic losses, infrastructure insurance claims, or outage reports—to form a comprehensive view of infrastructure vulnerability and damages.

Beyond historical analysis, this dataset also offers 14 CMIP6-based future projections (SSP245 and SSP585), enabling researchers and practitioners to estimate potential climate-driven shifts in hazard frequency and intensity. Users can draw on multiple models to construct ensemble (or “stochastic”) scenarios that capture uncertainty in future climate outcomes. While the 0.25° resolution and daily/sub-daily granularity are well-suited to analyses that require fine-scale details, users with broader objectives or limited computational resources can aggregate data to coarser spatial or temporal scales.

Although developed largely for infrastructure applications, this dataset can also support a wide range of other efforts, including community disaster preparedness evaluations, commercial or industrial site planning, and climate-related public health or environmental justice studies. Currently, data coverage is limited to the contiguous United States due to constraints in observational and reanalysis inputs. Nonetheless, users may extend the dataset to regions of interest outside the U.S. by applying the provided code, contingent on having appropriate observational or reanalysis data. Plans are underway to expand coverage globally and to update the dataset as new observational references, reanalysis products, or improved GCM simulations become available. Users should always account for possible model or observational biases when interpreting the data for their specific applications.

## Data Availability

Scripts used to generate the processed datasets and to create the figures in this article are openly shared at https://purl.stanford.edu/tx442fm4240. The repository also hosts the intermediate data files required to reproduce every plot. To obtain the original raw datasets (e.g., ERA5, NARR, NLDN, CMIP6 model outputs) that were ingested and processed in this study, please download them directly from the sources cited in the manuscript’s Methods sections.
